# Long-term kidney survival analyses in IgA nephropathy patients under steroids therapy: a case control study

**DOI:** 10.1186/s12967-015-0549-2

**Published:** 2015-06-06

**Authors:** Yanhong Yuan, Qin Wang, Zhaohui Ni, Xiajing Che, Liou Cao, Xinghua Shao, Minfang Zhang, Yuanyuan Xie, Chaojun Qi, Wenyan Zhou, Lei Tian, Shan Mou

**Affiliations:** Department of Nephrology, Molecular Cell Lab for Kidney Disease, Ren Ji Hospital, School of Medicine, Shanghai Jiao Tong University, 160 Pujian Road, Shanghai, 200127 China

**Keywords:** IgA nephropathy, Corticosteroids, Risk factors, Progression

## Abstract

**Background:**

Corticosteroids are preferred to treat patients with active IgA nephropathy (IgAN), and beneficial effects from the short-term use of corticosteroids have been confirmed. However, a large number of patients will progress to end-stage renal disease after a long time follow-up. This study aimed to evaluate kidney disease progression and risk factors on kidney survival in IgAN patients receiving steroids treatment.

**Methods:**

Two hundred biopsy-proven IgAN patients who received corticosteroid therapy were enrolled and followed for a median period of 63.33 months. Risk factors on kidney survival were retrospectively investigated by the Cox proportional hazards model.

**Results:**

Of the two hundred patients, twenty patients showed progression of renal impairment at the end of follow-up. The median and interquartile range values for initial serum creatinine were 89.2 and 68.08–121.35 µmol/L, respectively. Multivariate Cox regression analyses revealed that relapse, non-remission, time-averaged eGFR (TA-eGFR), and time-averaged serum albumin (TA-ALB) were independently associated with the kidney progression. Those with TA-ALB levels <35 g/L and TA-eGFR levels <60 mL/min/1.73 m^2^ were less likely to recover from kidney progression. Patients were more likely to show kidney function deterioration, when they had non-remission or relapse after corticosteroids treatment.

**Conclusion:**

This study demonstrated that relapse, non-remission, TA-eGFR, and TA-ALB could serve as independent predictors of long term prognosis of IgAN patients receiving corticosteroid therapy.

## Background

IgA nephropathy (IgAN) is the most commonly occurring glomerulopathy and very likely progresses to end-stage renal disease (ESRD) worldwide [1–4]. The pathogenesis of IgAN is still unclear, and no specific treatment is established [5]. Clinically, proteinuria is the most powerful predictor of poor kidney outcome, and reduction of proteinuria is associated with improved kidney survival [2, 3, 6–8]. Thus corticosteroids seem to be a good treatment, for its function of ameliorating proteinuria and protecting against kidney deterioration [9–11].

There has been widespread interest in corticosteroid therapy for IgAN. Studies have reported that prednisone can reduce urinary protein, protect kidney function, and delay the progress of IgAN [9–11]. Kidney disease: improving global outcomes (KDIGO) clinical practice guideline for glomerulonephritis suggest that patients with persistent proteinuria >1 g/day, despite 3–6 months of optimized supportive care [including angiotensin converting enzyme inhibitor (ACE-I) or angiotensin II receptor blocker (ARB) and blood pressure control], and GFR >50 mL/min per 1.73 m^2^, receive a 6-month course of corticosteroid therapy [12].

Beneficial effects from the short-term use of corticosteroids were reported in randomized control trial studies (RCTs) [13, 14]. However, in clinic, kidney function deterioration still occurred with a considerable amount of patients receiving prednisone. Moreover, the results of RCTs in adult patients are controversial, because of different outcomes consideration, small sample size or too short follow-up periods for a slowly progressing disease [15–17].

Therefore we retrospectively investigated the influence of clinical and laboratory potential risk factors on kidney survival in 200 patients receiving steroids treatment with IgAN by the Cox proportional hazards model.

## Methods

### Study population

IgA nephropathy patients were eligible for the study when the following criteria were satisfied: having received steroids therapy; age >18 years; follow-up duration >36 months. The exclusion criteria were as follows: 24-h urinary protein excretion (UPE) >3.5 g/day; serum albumin (ALB) <30 g/L; pregnancy; systemic inflammation, such as Henoch–Schönlein purpura; chronic advanced liver disease; or atypical forms of IgAN [acute kidney injury (AKI) associated with macroscopic hematuria and crescentic IgAN]. Two hundred patients with primary IgAN, who had been biopsied between January 2005 and December 2010 and received steroids treatment at the Department of Nephrology, Ren Ji Hospital, Shanghai, China, were included in this study. The study was approved by the Ethics Committee of Ren Ji Hospital, and all the participants gave written informed consent. All the kidney biopsy slides were reviewed by an experienced kidney pathologist.

### Study design

The influence of clinical parameters, histological grade and treatment on kidney survival was retrospectively examined. Clinical parameters and laboratory data used for analyses were obtained at the time of diagnosis of IgAN and during the follow-up, including age, gender, baseline systolic blood pressure (SBP) and diastolic blood pressure (DBP), medical history, medications, follow-up duration, time to remission, time to endpoint, and responsiveness to treatment, hemoglobin (Hb) (measured by cyanide methemoglobin method), UPE (measured by sulfosalicylic acid assay), hematuria (uRBC/HP), serum creatinine (SCr) (measured by ammonia iminohydrolase-PAP), serum uric acid (UA) (measured by a biosensor based on urate oxidase- peroxidase coupled enzyme system), blood urea nitrogen (BUN) (measured by urease coupling rate method), and ALB (measured by dye bromcresol green method).

As for the treatment, the effect of steroids therapy was examined. Any antihypertensive agent was permitted to control blood pressure during the follow-up. Using the modification of diet in kidney disease (MDRD) study equation calculated the estimated glomerular filtration rate (eGFR): eGFR (mL/min/1.73 m^2^) = 180 × [SCr (mg/dL)]^−1.154^ × (age)^−0.203^ × (0.742 if female) [18]. Based on the kidney disease outcomes quality initiative (K/DOQI) practice guidelines, chronic kidney disease (CKD) was classified. The mean arterial blood pressure (MAP) was defined as the DBP plus one-third of the SBP. Time-averaged values such as the time-averaged UPE (TA-UPE), the time-averaged ALB (TA-ALB), the time-averaged SCr (TA-SCr), and the time-averaged eGFR (TA-eGFR) were obtained just as previously described [19–21]. The kidney lesions were graded according to Lee’s classification at the time when the database was established [22].

After 6 months’ steroids therapy, the treatment response was evaluated. Complete remission (CR) was defined as a UPE <0.3 g/day, along with normalization of all biochemical findings and a lack of worsening of kidney function at the sixth month; partial remission (PR) was defined as at least a 50% reduction in UPE at the sixth month compared with baseline; no response (NR) was defined as a <50% reduction in UPE or an increase in UPE with or without kidney deterioration after receiving 6 months of therapy [23]. Relapse was defined as the reappearance of significant proteinuria, defined as >1.0 g/day and/or as a UPE increase of more than 50% [23, 24]. The end point of kidney survival was estimated by ESRD requiring hemodialysis therapy. The patients were classified with progression when their eGFR values decreased by more than 50% or when they reached ESRD during the follow-up period; the patients who exhibited stable kidney function, defined as an eGFR that remained within 50% of the initial value, were considered to be non-progression patients [25].

Most of the patients were treated according to the accepted standards at our center [12]. Long-term ACE-I or ARB treatment was recommended if there were no any contraindications. Corticosteroid was used in cases of massive proteinuria (>1 g/day). Corticosteroid regimens in patients with IgAN were 6-month regime of oral prednisone starting with 0.8–1 mg/kg/day for 2 months and then reduced by 0.2 mg/kg/day per month for the next 4 months. Other immunosuppressive agents were considered in patients with rapidly progressing kidney function decline.

### Statistics

Analyses of the data were carried out using SPSS software (version 13: SPSS, Chicago, IL, USA). The normally distributed variables were expressed as the mean ± SD and the non-parametric variables were expressed as the median and range. Cox’s proportional hazards models for estimating the hazard ratios and the 95% confidence intervals (CI) were used to identify the predictive factors for the development of IgAN progression. The multivariate models used a stepwise forward selection procedure based on a likelihood-ratio test with P > 0.10 for the removal and P < 0.05 for the entry of the variables. Kidney survival was estimated with the Kaplan–Meier method, and comparisons were performed using the log-rank test. Values of P less than 0.05 were considered statistically significant.

## Results

### Baseline characteristics of the study population

**Figure 1:**
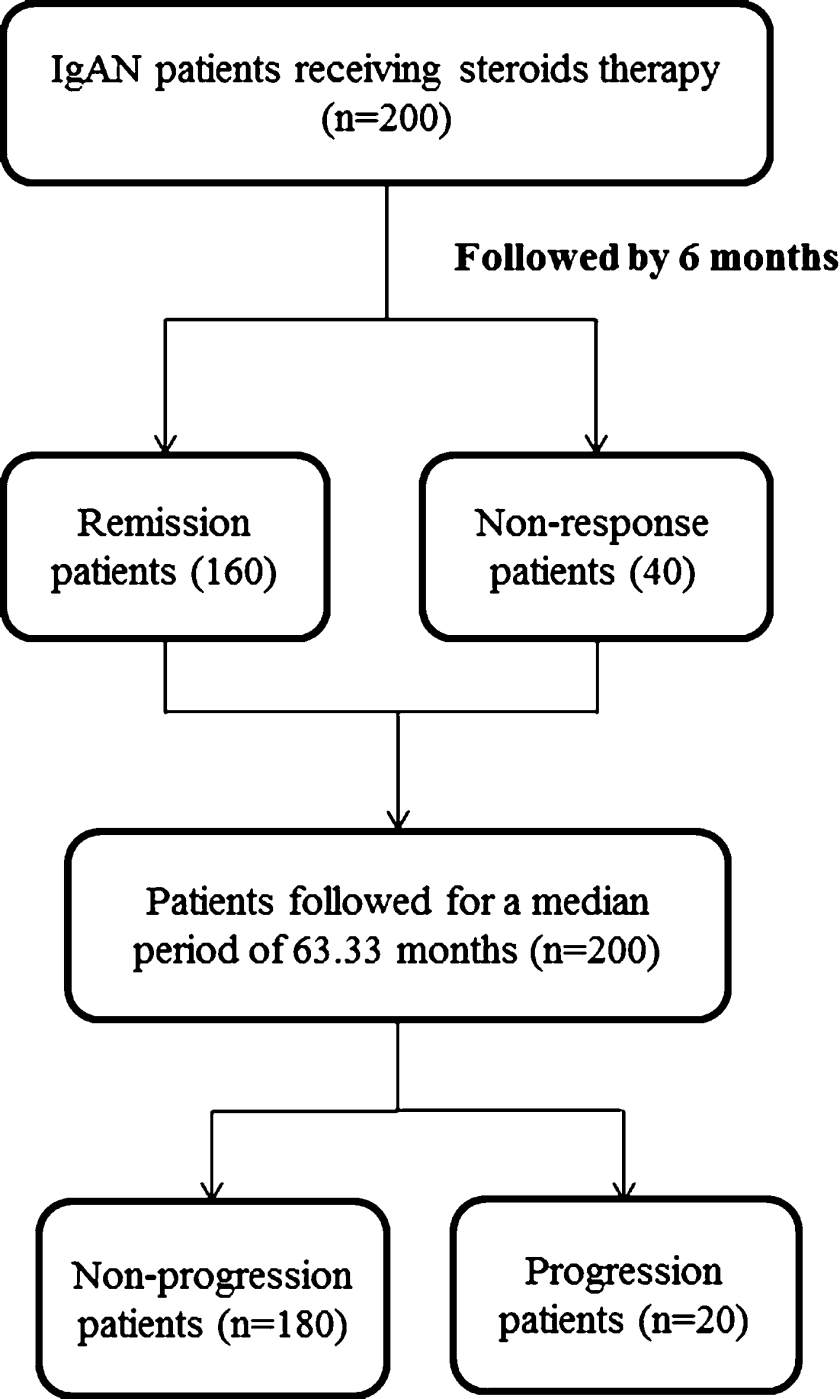
A flow diagram of the study.

From 2005 to 2010, the data of a total of 200 patients with IgAN who had received steroids treatment were utilized in the analyses (Figure 1). All the subjects were Chinese. The baseline characteristics of the patients were shown in Table 1. The median follow-up period was 63.33 months, ranging from 43.60 to 100.67 months. Twenty patients showed progression of renal impairment after steroid therapy at the end of the follow-up.

**Table 1:** Characteristics and clinical outcome in 200 IgAN patients receiving glucocorticoid medicine therapy

Characteristics	All included patients (N = 200)	Non-progression patients (N = 180)	Progression patients (N = 20)
Baseline			
Age (years)	36.99 ± 11.33	36.20 ± 10.42	43.93 ± 16.34
Gender: female, n (%)	113 (56.5)	100 (55.60)	13 (64.30)
SBP (mm Hg)	124.33 ± 17.11	124.08 ± 16.08	126.43 ± 24.99
DBP (mm Hg)	81.08 ± 11.10	81.33 ± 10.89	78.93 ± 13.04
SCr (µmol/L)	89.2 (68.08–121.35)	88.25 (67.35–117.70)	108.95 (84.80–139.40)
eGFR (mL/min/1.73 m^2^)	70.98 (51.91–95.93)	72.84 (52.93–96.39)	53.36 (46.55–62.35)
BUN (mmol/L)	5.98 (4.60–7.80)	5.90 (4.60–7.40)	6.78 (5.46–8.94)
UA (µmol/L)	363.00 (311.65–427.50)	359.00 (310.00–422.00)	380.50 (347.00–436.00)
Hb (g/L)	131.00 (120.00–143.00)	132.50 (120.00–144.00)	124.00 (121.00–132.00)
ALB (g/L)	38.20 (34.10–40.80)	38.40 (34.60–41.10)	33.75 (30.20–38.60)
UPE (g/day)	2.27 (1.35–3.41)	2.14 (1.34–3.14)	3.08 (2.27–3.84)
uRBC/HP	26.20 (8.03–67.48)	24.10 (7.80–66.55)	31.00 (12.30–51.70)
CKD stage, n (%)			
Stage I	67 (33.5)	63 (35.0)	4 (20.0)
Stage II	60 (30.0)	57 (31.6)	3 (15.0)
Stage III	68 (34.0)	55 (30.6)	13 (65.0)
Stage IV–V	5 (2.5)	5 (2.8)	0 (0)
FSGS, n (%)	112 (56.0)		
Renal biopsy Lee’s classification, n (%)			
Grade I	4 (2.1)	3 (1.7)	1 (5.0)
Grade II	4 (2.1)	2 (1.1)	2 (10.0)
Grade III	115 (57.4)	103 (57.2)	12 (60.0)
Grade IV	68 (34.0)	65 (36.1)	3 (15.0)
Grade V	9 (4.3)	7 (3.9)	2 (10.0)
Follow-up			
Remission, n (%)	160 (80)	152(84.4)	8 (40.0)
Complete remission, n (%)	71 (35.4)	68 (37.8)	3 (15.0)
Partial remission, n (%)	89 (44.6)	84 (46.7)	5 (25.0)
Non-response, n (%)	40 (20.0)	28 (15.6)	12 (60.0)
Relapse, n (%)	35 (17.3)	24 (13.3)	11 (55.0)
Progression, n (%)	20 (10)		
Length of follow-up (months)	68.33 (43.60–100.67)	67.33 (42.93–98.77)	71.42 (38.37–92.83)
ALB (g/L) at month 3	39.45 (35.80–43.03)	40.00 (35.90–43.30)	37.50 (35.00–40.00)
ALB (g/L) at month 6	41.50 (37.50–44.15)	42.20 (38.10–45.00)	36.75 (35.20–40.00)
TA-ALB (g/L)	40.93 (38.05–44.14)	42.48 (38.75–44.65)	37.28 (36.40–39.01)
SCr (µmol/L) at month 3	88.10 (71.60–105.00)	87.30 (71.60–104.80)	101.55 (78.80–112.20)
SCr (µmol/L) at month 6	80.00 (64.70–104.50)	79.00 (64.70–100.60)	97.85 (78.00–128.50)
TA-SCr (µmol/L)	85.24 (64.81–106.75)	83.73 (64.71–103.46)	99.34 (77.36–111.91)
eGFR (mL/min/1.73 m^2^) at month 3	70.34 (56.97–98.72)	73.67 (58.04–98.72)	61.98 (48.87–81.43)
eGFR (mL/min/1.73 m^2^) at month 6	80.02 (58.05–103.28)	81.08 (62.52–103.38)	58.68 (52.57–82.39)
TA-eGFR (mL/min/1.73 m^2^)	76.93 (58.30–103.33)	80.61 (59.49–104.98)	58.74 (52.66–83.32)
UPE (g/day) at month 3	0.79 (0.36–1.94)	0.73 (0.36–1.57)	1.26 (0.60–2.52)
UPE (g/day) at month 6	0.63 (0.21–1.51)	0.60 (0.19–1.39)	1.36 (0.72–2.29)
TA-UPE (g/day)	0.89 (0.51–1.75)	0.83 (0.49–1.69)	2.11 (0.82–2.36)

The mean age at biopsy was 36.99 ± 11.33 years. There were 87 men and 113 women. The systolic or diastolic blood pressure was 124.33 ± 17.11 mmHg and 81.08 ± 11.10 mmHg, respectively. The median and interquartile range (IQR) values for initial SCr were 89.2 and 68.08–121.35 µmol/L, respectively. Sixty-seven patients were classified in CKD stage I, 60 in stage II, 68 in stage III and 5 in stage IV–V. The median and IQR values for the initial ALB were 38.20 and 34.10–40.80 g/L, respectively. The median and IQR values for the initial proteinuria were 2.27 and 1.35–3.41 g/day, respectively. One hundred and eighty-three patients were classified in histological Lee’s grade III–IV.

### Characteristics of the study population during follow-up

The characteristics of the patients during the follow-up were shown in Table 1 as well, among which the median and IQR values for TA-ALB were 40.93 and 38.05–44.14 g/L, respectively. The median and IQR values for TA-eGFR were 76.93 and 58.30–103.33 mL/min/1.73 m^2^, respectively. The median and IQR values for TA-UPE were 0.89 and 0.51–1.75 g/day, respectively.

Finally, a total of 160 patients with IgAN achieved remission within 6 months of initiation of steroids therapy. The remaining 40 patients (20.0%) exhibited a minimal response or NR. Additionally, 17.3% of the remission patients experienced relapse during the follow-up. During the median follow-up period of 63.33 months, 10.0% of the 200 included patients met the criteria of progression in this study.

### Risk factors for the development of kidney progression in IgAN patients receiving steroids treatment

Both univariate and multivariate Cox analyses were performed to evaluate the impact of the potential predictors on kidney progression in IgAN patients receiving steroids treatment. As shown in Table 2, age, baseline ALB, baseline eGFR, response to therapy (non-remission vs remission and relapse vs non-relapse), ALB at month 6,UPE at month 3,UPE at month 6, TA-ALB, TA-eGFR, and TA-UPE were notably associated with the risk of kidney progression in the univariate analyses.

**Table 2:** Factors that were found to affect the long-term prognosis in IgAN patients receiving glucocorticoid medicine therapy in the univariate COX regression analyses

Characteristics	Univariate analysis		
	HR	95% CI	P value
Baseline			
Age (years)	1.063	1.014–1.115	0.011
ALB (g/L)	0.929	0.869–0.994	0.033
eGFR (mL/min/1.73 m^2^)	0.976	0.953–1.000	0.047
uRBC/HP			0.975
Renal biopsy Lee’s classification, n (%)			
Grade I			
Grade II			
Grade III			
Grade IV			0.596
Follow-up			
Length of follow-up (months)			0.684
Non-remission (vs remission)	5.877	2.031–17.006	0.001
Relapse (vs non-relapse)	12.602	1.443–110.058	0.022
ALB g/L at month 6	0.844	0.761–0.935	0.001
UPE at month 3 (g/day)	1.000	1.000–1.001	0.016
UPE at month 6 (g/day)	1.000	1.000-1.001	0.043
uRBC/HP at month 3			0.961
uRBC/HP at month 6			0.574
TA-ALB (g/L)	0.778	0.663–0.913	0.002
TA-eGFR (mL/min/1.73 m^2^)	0.978	0.957–1.000	0.048
TA-UPE (g/day)	1.000	1.000–1.001	0.046

Those factors that were significantly correlated with progression on the basis of univariate analyses were further evaluated by the multivariate analyses. Three multivariate Cox regression models were created, as shown in Table 3. In model 1, higher TA-ALB and higher TA-eGFR reduced the risk of occurring kidney function deterioration independently. As for the steroids treatment, hazard ratio (HR) for kidney progression of non-remission patients after steroids therapy increased significantly compared to the remission group (HR = 5.877, 95% CI = 2.031–17.006, P = 0.001). These findings were still observed in model 2 after adjusting for the effects of age, gender, eGFR, ALB, and UPE (HR = 4.995, 95% CI = 1.436–17.365, p = 0.011). In the adjusted model, individuals who experienced relapse had a greater risk of kidney progression compared with non-relapse individuals (HR = 12.629, 95% CI = 1.446–110.320, p = 0.021) (Table 3, model 3).

**Table 3:** Factors that were found to affect the long-term prognosis in IgAN patients receiving glucocorticoid medicine therapy in the multivariate COX regression analyses

Characteristics	Univariate analysis			Multivariate analysis		
	HR	95% CI	P value	HR	95% CI	P value
Model 1 (n = 200)						
Age (years)	1.063	1.014–1.115	0.011			0.20
Gender			0.48			0.42
TA-eGFR (mL/min/1.73 m^2^)	0.978	0.957–1.000	0.048	0.971	0.945–0.998	0.03
TA-ALB (g/L)	0.778	0.663–0.913	0.002	0.772	0.636–0.937	0.008
UPE at month 3 (g/day)	1.000	1.000–1.001	0.016			0.40
Model 2 (n = 200)						
Age (years)	1.063	1.014–1.115	0.011			0.25
Gender			0.48			0.32
Non-remission (vs remission)	5.877	2.031–17.006	0.001	4.995	1.436–17.365	0.011
TA-eGFR (mL/min/1.73 m^2^)	0.978	0.957–1.000	0.048	0.962	0.933–0.992	0.013
TA-ALB (g/L)	0.778	0.663–0.913	0.002	0.806	0.673–0.966	0.019
UPE at month 3 (g/day)	1.000	1.000–1.001	0.016			0.99
Model 3 (n = 160)						
Age (years)	1.063	1.014–1.115	0.011			0.52
Gender			0.48			0.13
Relapse (vs non-relapse)	12.602	1.443–110.058	0.022	12.629	1.446–110.32	0.021
TA-eGFR (mL/min/1.73 m^2^)	0.978	0.957–1.000	0.048			0.06
TA-ALB (g/L)	0.778	0.663–0.913	0.002			0. 12
UPE at month 3 (g/day)	1.000	1.000–1.001	0.016			0.28

**Figure 2:**
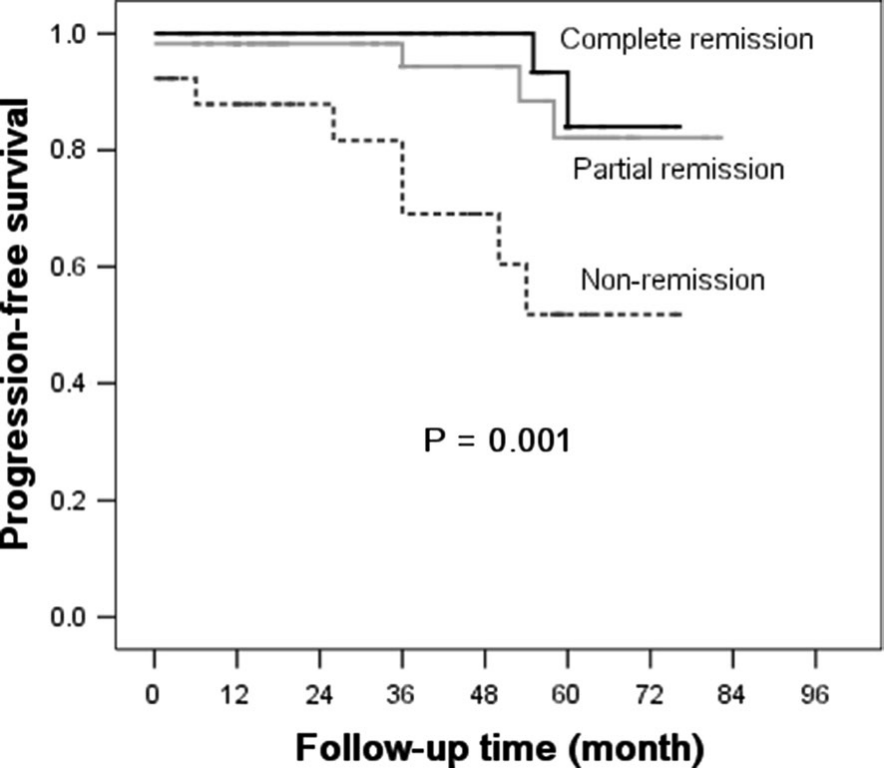
Progression-free survival analysis according to remission.

**Figure 3:**
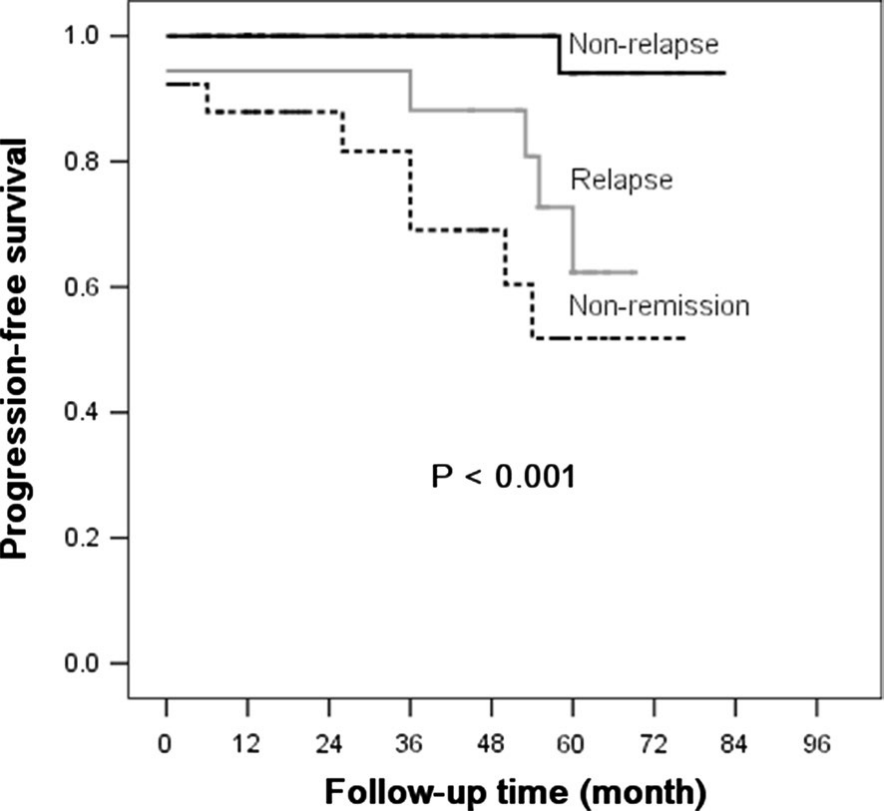
Progression-free survival analysis according to relapse.

**Figure 4:**
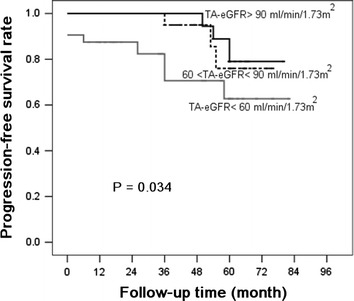
Progression-free survival analysis according to TA-eGFR.

**Figure 5:**
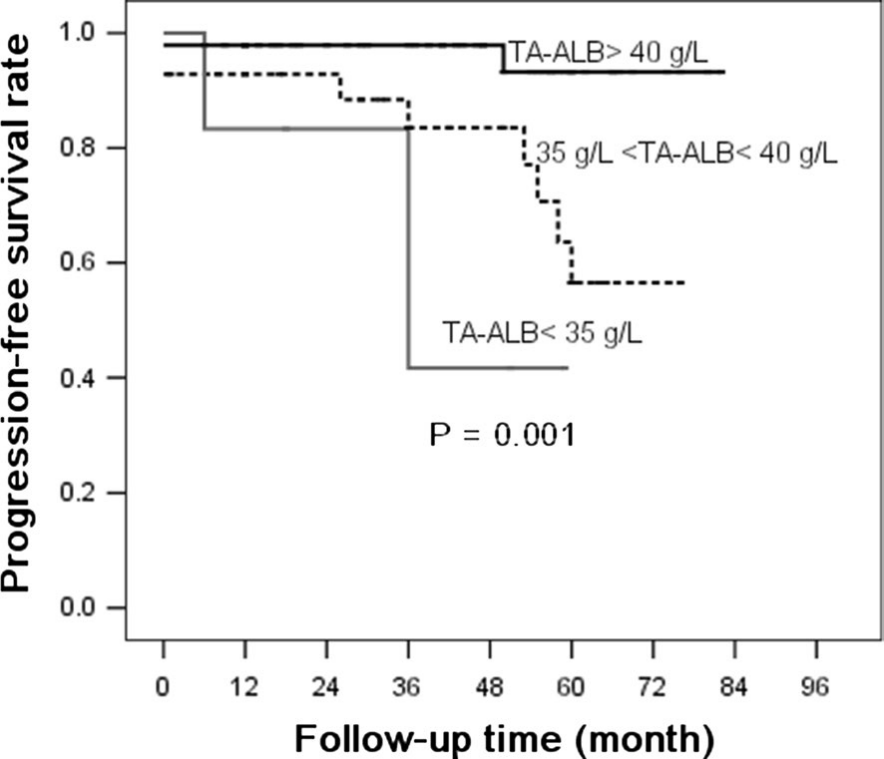
Progression-free survival analysis according to TA-ALB.

Based on the Kaplan–Meier analyses, the actual kidney survival according to the potential risk factors were plotted in Figures 2, 3, 4 and 5. As illustrated in Figure 2, remission patients (CR or PR patients) had significantly longer progression-free times compared with the non-remission patients (P < 0.05). The patients who experienced relapse after achieving remission, had an unfavorable survival rate of non-progression (Figure 3). The relationship between the TA-ALB values and kidney outcomes was dramatically altered at levels of 40 and 35 g/L. And the relationship between the TA-eGFR levels and kidney outcomes was altered at levels of 90 and 60 mL/min/1.73 m^2^ (Figures 4, 5).

## Discussion

Long-term prognosis of IgAN was highly variable: ESRD occurred in 5–25% of cases within 10 years [2, 3, 26] and in 25–50% within 20 years [3]. There were a variety of studies having confirmed the effect of steroids treatment on amelioration of the clinical course of IgAN [14, 15]. In clinical practice, beneficial effects from the short-term use of corticosteroids in the small sample of patients were clear and definite. However, the long-term efficacy of steroids therapy in IgAN had barely been testified,and a number of IgAN patients progressed to ESRD even having received steroids therapy. RCT studies have confirmed that 50% increase in plasma creatinine from baseline occurred in about 10% of IgAN patients within 4–5 years of starting steroids treatment [10, 27, 28]. Similarly, kidney progression occurred in 20/200 (10%) included patients of this study. Thus the aim of this study was to identify risk factors for kidney function deterioration in a cohort of IgAN patients receiving steroids treatment.

Among the clinical and laboratory indicators of the disease, persistent and severe proteinuria was identified as the most important predictor of a poor outcome [2, 3, 6–8] and that its reduction correlates with better kidney function. A long-term RCT of 86 adult IgAN patients evaluating corticosteroids effectiveness in IgA nephropathy showed that, in addition to steroids, a reduction in proteinuria levels after 6 months, and no increase in proteinuria during follow-up all were independent predictors of a beneficial outcome [13].

Results of this study demonstrated that proteinuria at month 3 and month 6 was a strong related factor of kidney function decline in patients with IgAN according to univariate analysis. After a multivariate Cox analysis, we were unable to provide clear evidence that proteinuria was associated with kidney outcomes. This parameter did not independently contribute to the risk in the multivariate models.

Instead, the adjusted multivariate Cox analysis model revealed that non-remission state was associated with a 3.995 folds of increase in the risk of kidney progression, confirming the importance of proteinuria reduction in retarding IgAN progression. A recent multi-center RCT assessing whether the combination of prednisone and ramipril was more effective than ramipril alone in patients with proteinuric IgAN, revealed that a decrease in 24-h proteinuria <1 g was observed in 36/48 (75.0%) patients of the combination therapy group and in 33/49 (67.3%) patients of the monotherapy group at 6 months of follow-up [14]. In this study, CR and PR were achieved in 71 (35.4%) and 89 (44.6%) patients, respectively. The remaining 20.0% patients presented persistent proteinuria.

Relapse of proteinuria was commonly seen in IgAN patients. In this study, 28/160 (17.3%) patients appeared relapse after achieving remission, and individuals who experienced relapse had a significantly increased risk of kidney progression after adjusting for the effects of age, gender, eGFR, ALB, and UPE. This result verified previous viewpoint as well [13].

Serum albumin was a biomarker of nutritional status and inflammation [29]. In our previous study, we had demonstrated that the TA-ALB value was an independent predictor of kidney progression in IgAN patients who had achieved remission after treatment [19]. In the present study, baseline ALB, ALB at month 6 and TA-ALB correlated with kidney function decline in patients receiving steroids therapy by the univariate analysis. This result was in accordance with findings observed across multiple cohorts [20, 30–32].

Damaged baseline eGFR was a traditional risk factor for kidney progression. Similar to prior reports [3, 20, 33–35], we confirmed that the baseline eGFR and TA-eGFR were associated with kidney progression among IgAN patients receiving steroids treatment. Uncontrolled hypertension during follow-up is associated with greater proteinuria and predicts a faster GFR decline. Patients included in our study were monitored closely and under strict blood pressure control. Most patients had been kept within the normal blood pressure range after ACEIs/ARBs treatment during long term follow-up. Some studies had identified that elder patients would progressed to ESRD more promptly [2, 36–38]. Our multivariate analysis also suggested that older age at diagnosis was a strong predictor of kidney progression among patients having received steroids therapy.

This is a study to use a cohort of IgAN patients having received steroids treatment after diagnose to assess kidney outcomes and the related risk factors for kidney progression. Our study may help explain the reason of a large number of patients receiving prednisone still progressed to ESRD. This study is unique in that it identified predictors for clinical outcomes among a pool of IgAN patients receiving steroids therapy. The strengths of this study also included a relative large number of IgAN patients, a uniform therapy strategy, and the robust database. Using this robust database, we examined the impact that clinical patterns have on progression to ESRD. Several shortcomings of this study existed. This study was a retrospective study and most of the patients recruited came from the southern regions of China. Therefore, the analyses had an inherent selection bias and limited representation. Thus, more studies are required to evaluate the progression of IgAN in patients under steroids treatment regimen in the future.

## Conclusions

In summary, our data clearly showed that relapse, non-remission, TA-eGFR, and TA-ALB were associated with renal outcomes. Furthermore, these characteristics could serve as independent predictors of long term prognosis of IgAN patients receiving corticosteroid therapy.
